# Bone Morphogenetic Protein 6 Polymorphisms Are Associated with Radiographic Progression in Ankylosing Spondylitis

**DOI:** 10.1371/journal.pone.0104966

**Published:** 2014-08-14

**Authors:** Young Bin Joo, So-Young Bang, Tae-Hwan Kim, Seung-Cheol Shim, Seunghun Lee, Kyung Bin Joo, Jong Heon Kim, Hye Joon Min, Proton Rahman, Robert D. Inman

**Affiliations:** 1 Department of Rheumatology, Hanyang University Hospital for Rheumatic Diseases, Seoul, Republic of Korea; 2 Division of Rheumatology, Daejeon Rheumatoid & Degenerative Arthritis Center, Chungnam National University Hospital, Daejeon, Republic of Korea; 3 Department of Radiology, Hanyang University Hospital for Rheumatic Diseases, Seoul, Republic of Korea; 4 Department of Orthopedics, Hanyang University Hospital for Rheumatic Diseases, Seoul, Republic of Korea; 5 Department of anthropology, Cornell University, Ithaca, New York, United States of America; 6 Department of Rheumatology, Memorial University, St. Clare’s Mercy Hospital, St. John’s, Newfoundland, Canada; 7 Division of Rheumatology, University of Toronto, Toronto Western Hospital, Toronto, Ontario, Canada; University of Texas Health Science Center at Houston, United States of America

## Abstract

**Background and Object:**

Nearly 25 genetic loci associated with susceptibility to ankylosing spondylitis (AS) have been identified by several large studies. However, there have been limited studies to identify the genes associated with radiographic severity of the disease. Thus we investigated which genes involved in bone formation pathways might be associated with radiographic severity in AS.

**Methods:**

A total of 417 Korean AS patients were classified into two groups based on the radiographic severity as defined by the modified Stoke’ Ankylosing Spondylitis Spinal Score (mSASSS) system. Severe AS was defined by the presence of syndesmophytes and/or fusion in the lumbar or cervical spine (n = 195). Mild AS was defined by the absence of any syndesmophyte or fusion (n = 170). A total of 251 single nucleotide polymorphisms (SNPs) within 52 genes related to bone formation were selected and genotyped. Odds ratios (OR) and 95% confidence interval (95% CI) were analysed by multivariate logistic regression controlling for age at onset of symptoms, sex, disease duration, and smoking status as covariates.

**Results:**

We identified new loci of bone morphogenetic protein 6 (*BMP6*) associated with radiographic severity in patients with AS that passed false discovery rate threshold. Two SNPs in *BMP6* were significantly associated with radiologic severity [r*s270378* (OR 1.97, p = 6.74×10^−4^) and *rs1235192* [OR 1.92, p = 1.17×10^−3^]) adjusted by covariates.

**Conclusion:**

This is the first study to demonstrate that *BMP6* is associated with radiographic severity in AS, supporting the role wingless-type like/BMP pathway on radiographic progression in AS.

## Introduction

Ankylosing spondylitis (AS) is a chronic inflammatory disease that preferentially affects the axial structures causing spinal ankylosis [1]. The process of ankylosis is closely associated with permanent work disability as well as decreased quality of life [2]

AS is a highly heritable (>90%), and human leukocyte antigen B27 (HLA-B27) is the strongest genetic association with AS, with >80% of patients being positive for HLA-B27 [3], [4]. However, HLA-B27 contributes only 16–50% of genetic risk [5], reflecting the fact that other non-HLA-B27 variants likely influence disease susceptibility. Recently, the International Genetics of Ankylosing Spondylitis Consortium confirmed the association of 25 loci at genome-wide significance in addition to HLA-B27. This included 12 of the 13 previously reported loci associated with AS in Europeans and 13 additional loci [1].

Although radiologic severity is also largely heritable (>60%) [6], there has been limited studies addressing the genetic influence on severity in contrast to studies of susceptibility to AS. A few studies confirmed the positive association with severity of AS, which have reported that that large multifunctional peptidase (LMP) 2, major histocompatibility complex and in endoplasmic reticulum aminopeptidases (ERAP) 1 have been reported to affect radiographic severity in AS [7]–[11]. These studies generally used candidate susceptibility gene of AS for analysis of possible associated genetic markers with severity. Two studies did not identify any significant genetic markers showing significant association with radiographic severity in AS [12], [13].

Underlying mechanism of new bone formation in AS remain incompletely understood. Current concepts propose a complex interaction between chronic inflammation and wingless-type like (WNT) pathway [14]. A recent study demonstrated that uncoupled interaction of WNT pathway with inflammation may play a key role in the development of new bone formation in AS. The effect of anti-tumor necrosis factor alpha agents on radiographic progression in AS has led to differing conclusions [15], [16]. The effect of increased C-reactive protein (CRP) or erythrocyte sedimentation rate (ESR) on structural change in AS is also inconclusive; some report its positive relationship [17], [18], but others are not, especially in longstanding AS [19], [20]. Some studies showed Dickkopf-1 and sclerostin is associated with radiographic severity independently inflammation, implicating complex molecular mechanisms, which can directly inhibit or enhance the WNT pathway, and which could be significantly impacting new bone formation in AS [21]–[23].

Based on these data, we hypothesized that genetic factors related to bone formation could be responsible for differential radiographic severity amongst AS. To test this hypothesis, we investigated the potential association of radiographic severity with the polymorphisms of genes involved in bone formation in Korean patients with AS.

## Materials and Methods

### Study Population and Clinical Data

We included a total of 417 patients with AS who are all of Korean ethnicity, recruited from the Hanyang University Hospital for Rheumatic Disease. All patients with AS satisfied the 1984 modified New York criteria for AS [24]. Clinical data collected included age, gender, age at disease onset, which means the onset age of axial symptoms, disease duration, smoking status, nonsteroidal anti-inflammatory drugs (NSAIDs) dose and duration used, HLA-B27 positivity, baseline ESR, and CRP. NSAIDs used was scored by the method which Dougados et al. suggested [25].

### Radiographic Scoring

The modified Stoke Ankylosing Spondylitis Spinal Score (mSASSS), which is considered the standard for quantification of chronic spinal changes in AS, was used for assessing radiographic severity. [26], [27] In mSASSS, scoring evaluates the anterior radiographic changes of the lumbar spine and cervical spine in lateral radiographic view: 0 - normal, 1 - erosion, squaring, or sclerosis, 2 -syndesmophyte or 3– bridging syndesmophyte (maximum 72) [28] In the cases with less than 3 vertebral site missing, the missing scores were substituted by the mean score of the vertebra of the same spinal segment of the patients. Two expert radiologists (SL, KBJ) scored independently. Then, discordant scores were reevaluated by both readers. Their interclass and intraclass correlation coefficients were 0.95 and 0.97, respectively.

### Severity Classification

The patients with AS are classified into two groups-mild or severe-based on the radiographic severity as follows. Within the measurement error of mSASSS, that scores of 1 is intermediate, and perhaps of indeterminate significance. Also, since a syndesmophyte at only 1 level can be seen in other state than AS, severe AS was defined by three or more syndesmophytes and/or fusion at the lumbar spine or cervical spine. Mild AS was defined by the absence of any syndesmophyte. Patients who had only 1 or 2 syndesmophytes or fusion were excluded from the analysis to allow a clear differentiation of severity between mild and severe.

### Genotyping

**Figure 1:**
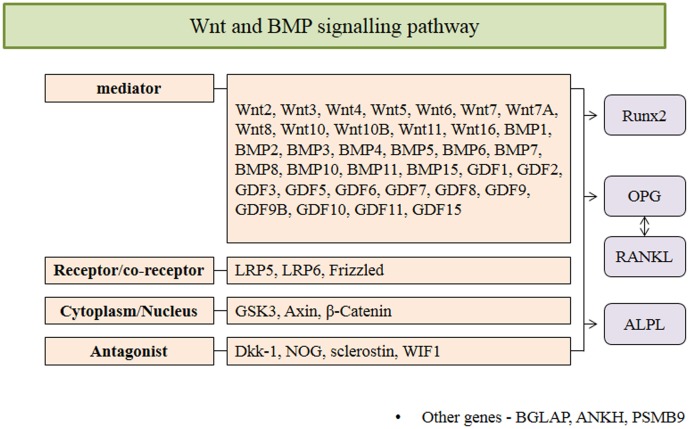
52 Gene lists analyzed in our study. Genes were selected from public databases including the SNP database of the National Centre for Biotechnology Information (NCBI; http://www.ncbi.nlm.nih.gov/SNP/) and the International HapMap Project (http://www.hapmap.org/).

In this study, 52 candidate genes (see **Figure 1** and **Table S1**) associated with involved in bone formation pathways were selected from public databases including the SNP database of the National Centre for Biotechnology Information (NCBI; http://www.ncbi.nlm.nih.gov/SNP/) and the International HapMap Project (http://www.hapmap.org/). SNP genotyping using the Sequenom MassARRAY® system (iPLEX GOLD) was performed according to the manufacturer’s instructions (Sequenom, San Diego, CA, USA). Briefly, PCR and single-base extension (SBE) primers were designed using MassARRAY assay design software (Sequenom, San Diego, CA, USA). Manufacturer’s instructions for the multiplex reaction were followed for the PCR amplification, the shrimp alkaline phosphatase (SAP) enzyme treatment, the SBE reactions using an iPLEX GOLD assay, and the clean-up with a resin kit (Sequenom, San Diego, CA, USA). The multiplex assays were designed using Sequenom’s Assay Design Suite 1.0. Only 251 SNPs of 52 genes were genotyped due to problems inherent with designing multiplex reactions. PCR and SBE primers sequences and all protocols are available upon request. Reaction products were dispensed onto a SpectroCHIP bioarray (Sequenom, San Diego, CA, USA) using a MassARRAY nanodispenser (Sequenom, San Diego, CA, USA) and assayed on the MassARRAY platform (Sequenom, San Diego, CA, USA). Differences in mass were detected with matrix-assisted laser desorption/ionization time-of-flight mass spectrometry (MALDI-TOF MS). MassARRAY Workstation software was used to process and analyse the iPLEX SpectroCHIP bioarray. Typer Analyzer software was used to analyse all genotypes obtained from the assays.

### Statistical Analysis

We eliminated SNPs that had insufficient call rates (<90% and minor allele frequency <1%) in cases and controls, Hardy-Weinberg disequilibrium in controls (*p*<1×10^−5^), and samples that were less than 90% sequenced. To determine the association of respective SNPs with radiologic severity, odds ratio (OR) and 95% confidence interval were calculated using logistic analysis (allelic model), controlling for age of disease onset, sex, disease duration, and smoking status. Given the large number of tests, there was high potential for false discovery. Thus, we used a false discovery rate (FDR) method to control the error inherent in multiple comparisons [29]. The association of genotype of significant genes with total mSASSS was analyzed using Kruskal-Wallis test with Mann Whitney P. Statistical analyses were conducted in PLINK v1.07 and SPSS17 software (Chicago, IL, USA).

### Ethics Statement

The Institutional Review Board of Hanyang University approved the protocol. All patients gave written informed consent.

## Results

### Clinical Characteristics of Patients with AS

In this study, we included the AS patient with longstanding disease; the mean disease duration from symptoms onset to the time when radiograph was taken was 14.1±6.8 years, and the mean score of mSASSS was 23.1±21.9 **(** **Table 1** **)**. After excluding the patients who had only 1 or 2 syndesmophyte or fusion, a total of 365 patients were classified into 2 groups such as severe (n = 195) or mild AS (n = 170). Patients with severe AS were older at onset of symptoms (41.6±8.1 vs 33.0±6.8), had higher percentage of males (97.6 vs 89.2) and smokers (74.0 vs 51.8), and had longer disease duration (16.7±7.3 vs 11.9±5.4) compared with those with mild AS. The percentage of HLA-B27, NSAID used, ESR, and CRP were not significantly different between the two groups. Total mSASSS (range 0–72) was 41.5±19.7 in patients with severe AS, and 7.2±3.6 in that with mild AS (*p*<0.001).

**Table 1:** Clinical characteristics of study cohort.

	All patients (n = 365)	Severe AS (n = 195)	Mild AS (n = 170)	*p*
Age at onset, year	37.0±8.6	41.6±8.1	33.0±6.8	<0.001
Male	340 (93.2)	174 (89.2)	166 (97.6)	0.001
Disease duration, year	14.1±6.8	16.7±7.3	11.9±5.4	<0.001
Smoker (n = 322)	201 (62.4)	114 (74.0)	87 (51.8)	<0.001
HLA-B27 (n = 344)	334 (97.1)	151 (98.7)	183 (95.8)	0.195
ESR, mm/hr (n = 197)	20.5±23.9	23.2±26.4	18.1±21.4	0.135
CRP, mg/dL (n = 194)	1.2±2.2	1.4±2.7	1.1±1.6	0.381
mSASSS, range 0–72				
Cervical spine	12.2±11.0	19.4±12.5	6.0±2.7	<0.001
Lumbar spine	10.8±13.1	21.9±11.5	1.2±2.1	<0.001
Total	23.1±21.9	41.5±19.7	7.2±3.6	<0.001
*Continuous NSAID intake (n = 161)	44 (27.3)	24 (33.3)	20 (22.5)	0.124

Data were shown to mean ± SD or n (%).

*Continuous NSAID intake was defined as 70 or more the score. AS; ankylosing spondylitis, HLA-B27: human leukocyte antigen-B27; ESR: erythrocyte sedimentation rate; CRP: C-reactive protein; Msasss: modified stokes AS spine score; NSAID: non-steroidal anti-inflammatory drugs.

### Associations of SNPs Related to Bone Formation Mechanism with Radiologic Severity

To control the clinical differences between two groups, logistic regression analysis was adjusted for age at onset of symptoms, sex, disease duration, and smoking status. Among 52 genes analyzed, only BMP6-related SNPs were associated with radiographic severity **(** **Table 2** **and Table S2)**. SNP *rs270378* of BMP6 showed the strongest association with severe AS (OR 1.97, p = 6.74×10^−4,^ Table 2). *rs1235192* of BMP6 was also associated with severe AS (OR 1.92, p = 1.17×10^−3^), although these SNPs did not reach significance after Bonferroni correction. These two SNPs were not in linkage disequilibrium (LD) (R2 = 0.004, D′ = 0.106, Distance: 104 kb).

**Table 2:** Significant association between polymorphism associated with bone formation in AS patients.

SNP	Gene	Chr	Position	Risk Allele	Allele Freq. (Cases)	Allele Freq. (Controls)	OR	95% CI	*p*	FDRthresholds
rs270378	BMP6	6	7762715	C	0.585	0.497	1.97	1.33 - 2.90	6.74×10^−4^	1.56×10^−3^
rs1235192	BMP6	6	7867046	G	0.716	0.606	1.92	1.30–2.86	1.17×10^−3^	1.61×10^−3^

AS: ankylosing spondylitis; SNP: single nucleotide polymorphism; Chr: chromosome; Freq: frequency; OR: odds ratio; CI: confidence interval; FDR: false discovery rate; BMP6: bone morphogenetic protein 6.

### Association of Allele Frequency (Genotype) of BMP6 with Total mSASSS

**Figure 2:**
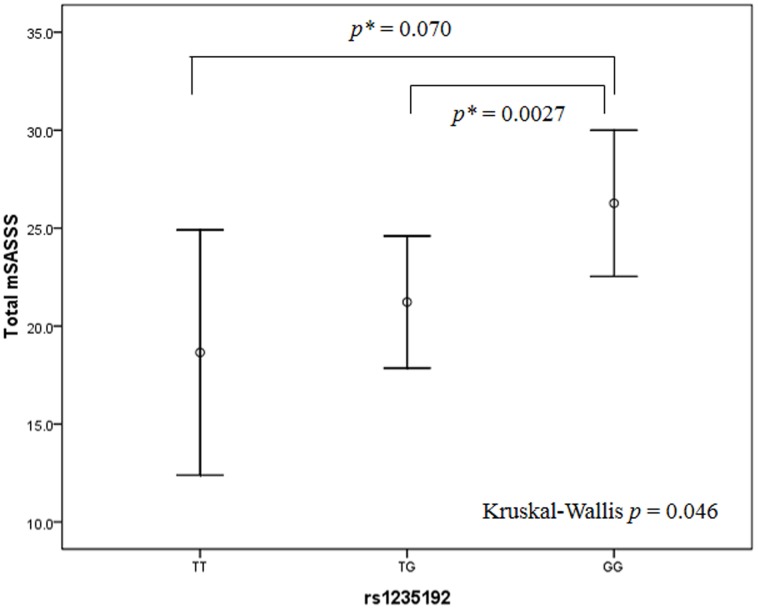
Association of genotype of BMP6 with total mSASSS. Data represent mean (95% CI). p = 0.046 by the Kruskal-Wallis test. *Mann Whitney P.

We looked at the trend of the allele with the actual mSASSS. In this study, it appears that the presence of risk allele in *rs1235192* was associated with markedly increased total mSASSS. As shown in **figure 2**, mSASSS in the group with G (TG or GG) was much higher than that in the group without it (p = 0.046). But the allele difference of SNP rs270378 for mSASSS was not statistically significant (P>0.05).

## Discussion

Our results highlight the role of certain biological pathways in the pathogenesis of AS. Recently, new hypothesis have been proposed that WNTs and BMPs are likely to play an important role in new bone formation in AS [14]. However, genetic studies that were examined this hypothesis are few, although genetic factors are likely playing a key role in defining new bone formation [14]. In this study, we specifically examined SNPs of genes related to bone formation, and most of which were related to WNTs and BMPs pathways. Among them, risk alleles at *rs270378* and *rs1235192* in *BMP6* were found to increase the risk of syndesmophyte formation. These relationships remained significant even after adjusted *P* value by FDR was applied to the multiple comparisons. We demonstrated for the first time that SNPs in *BMP6* likely play a contributory role in syndesmophyte formation or ankylosis in AS.

Recently, several genetic association studies were done to identify the risk variant for radiographic severity in AS. Haroon et al reported that *LMP2* variants in Caucasian AS affected baseline mSASSS, but not radiographic progression [7]. ERAP1 variants was reported to be associated with syndesmophyte formation in Taiwanese patients with AS, as reported by Wang et al [8]. This study compared the polymorphisms of genes between patients with at least one syndesmophyte vs those with no syndesmophytes. Two studies have suggested that prediction of radiographic severity was improved by genetic variants by showing prediction model of radiographic severity using the bath ankylosing spondylitis radiology index (BASRI) [10], [30].

Our results add to the knowledge about the genetic factors of AS radiographic severity in above results. The present study used the mSASSS to define severe AS, since the mSASSS had been accepted as the optimal method for quantification of chronic spinal changes [26] Other method such as BASRI is difficult to differentiate less or more severe spinal disease as its ceiling effect, But the mSASSS method could. The study addressed specifically severe cases, thereby eliminating cases of intermediate severity which may confound clear stratification for severity. There are reports of successfully identifying the risk variant using extremely high or low levels of interest [31], [32]. We also adjusted for smoking status, which is known to important environmental risk factor for radiographic progression, as well as age, sex, and disease duration. These points considered in our study may lead to accurate prediction of genetic markers of radiographic severity.

We found that the two (rs270378 and rs1235192) SNPs in BMP6 are associated with increased risk of syndesmophyte formation. These SNPs were not in LD (R2 = 0.004, D′ = 0.106, Distance: 104 kb). Interestingly, stronger effect on syndesmophytes was found in the group carrying double risk SNPs, rs270378 C and rs1235192 G (P = 3.48×10–4). There were two separate signals at this locus. The association at each SNP in the locus was with a common variant. All these findings suggest that these two SNPs confer the risk for syndesmophytes through independent contribution.

In this study, it appears that the presence of risk allele in rs1235192 was associated with markedly increased total mSASSS. As shown in figure 2, the mSASSS score in the group with G was much higher than that in the group without it (P = 0.046). But the allele difference of SNP rs270378 for mSASSS score was not statistically significant (P>0.05).

BMPs play an important role in bone morphogenesis and remodeling in health and disease. BMPs, including BMP6, are important in bone metabolism and can induce ectopic osteogenesis [33]. It has been known that *BMP6* messenger RNA is localized in hypertrophic cartilage [34], and *BMP6* has an important role in the maintenance and repair of human articular cartilage [35]. Polymorphisms in *BMP6* were independently associated with risk for sickle cell osteonecrosis [36], [37], pulmonary hypertension in sickle cell disease, [38] breast cancer growth and progression [39]. However, there was no report about variants in *BMP* genes and their association with new bone formation in AS.

In AS, two process of endochondral and direct bone formation contribute to ankylosis process. WNTs and BMP signaling play a role in endochondral bone formation and WNTs also play in direct bone formation [14]. In WNT pathway, WNT bind to low-density lipoprotein receptor-related protein 5 and 6 (LRP5 and 6) on mesenchymal cells followed by activation of intracellular β-catenin involve in bone formation proceeds. During this process, other key molecules such as BMP, axin, glycogen synthase kinase 3 beta (GSK3β), pronounced like the toy Frisbee (FRZB) interact each other to enhance or inhibit to bone formation [14]. WNT signaling elements such as Wnt3a and Wnt10b are associated with direct membranous bone formation, whereas, over-expression of β-catenin in late stage of chondrogenesis is associated with endochondral bone formation through stimulation of the chondrocytes maturation [40]. We investigated the polymorphisms in genes associated with WNT pathway but we did not identify the risk variant affecting the differential radiographic severity in AS.

BMPs are also important for signaling in bone formation process in AS. In the presence of BMPs, progenitor cells first differentiate into chondrocytes building a cartilaginous template that is subsequently replaced by bone [41]–[43]. Three specific BMPs have been studied in human AS. The levels of BMP2, BMP4, and BMP7 increased in AS patients with spinal fusion compared with patients without fusion [44]. However, these changes in BMP2 and BMP7 are not specific to AS, since BMP2 and BMP7 also increased in RA patients [45]. Until now, there is no report regarding to the level of BMP6 and its association with radiographic severity in human AS.

An Interesting observation on ankylosis in experimental models was seen in the study of Lories et al. [46]. They demonstrated that different BMPs are expressed during the process of ankylosis in male DBA/1 mice. BMP2 was induced in the early stage, BMP7 affects prehypertrophic chondrocytes, and BMP6 affects to hypertrophic chondrocytes in later stage. By immunohistochemistry staining, BMP6 was positive in hypertrophic chondrocyte-like cell showing later stage of endochondral bone formation in ankylosing enthesitis. This is interpreted as indicating that BMP6 is necessary to complete bone formation. This finding supports to our results that polymorphisms of BMP6 could affect to the bone formation, especially syndesmophyte formation in AS. However, further study investigating human histologic finding is needed to demonstrate the biologic role of BMP6 in syndesmophyte or ankylosis formation in human AS.

There is some limitation in this study. We used the cross-sectional data not longitudinal data. To determine the radiographic severity, it would be optimal to compare the radiographs between baseline and later follow-up. To address this in part, we selected primarily longstanding AS patients. The mean disease duration is 14.1±6.8 years, and more than 75% had disease duration of 10 years or more. Considering that significant radiographic progression commonly occurs in the first 10 years of disease [47] and that the strongest predictor of radiologic spinal progression is the presence of syndesmophytes at baseline [19], [48]–[50], mild AS patients who have no syndesmophyte over a course of 11.9±5.4 years of disease are likely to how minimal progression over time. In contrast, patients with severe AS who have already three more syndesmophytes at entry to the clinic are likely to show increased radiographic progression during further follow-up. We had not included functional data in the study. However, our result was supported by the experimental study of Lories et al discussed above [46]. As mentioned above, BMP6, which was associated with increased risk of development of syndesmophyte in our study, has been found in the early course of bone formation in animal immunohistochemistry study. However, future functional study in patients with AS is needed for better understanding of the role of BMP6 in bone formation. Finally, type 1 error of our result was controlled with the FDR method, not Bonferroni correction as our sample size was small. Despite the perception of small sample size, this is the largest racially and ethinically homogeneous AS population with mSASSS reported to date. Thus our results are meaningful and acceptable as type 1 error of result was controlled with the FDR method.

In summary, we show that certain BMP6 polymorphisms, especially *rs270378* and *rs1235192,* are possible risk factors for the development of syndesmophyte and ankylosis in AS. These variants could be excellent candidates for further investigation although replication in larger sample and in different ethnic groups is needed.

## Supporting Information

Table S1 This table contains the 366 single nucleotide polymorphisms (SNPs) analyzed in our study.
(DOCX)

Table S2 SNPs Association between polymorphism associated with bone formation in AS patients (p<0.05, adjusted by age at onset of symptom, sex, disease duration, and smoking).
(DOCX)
