# Supplementary material for: Bone Morphogenetic Protein 6 Polymorphisms Are Associated with Radiographic Progression in Ankylosing Spondylitis
Source: PLoS One. 2014 Aug 14;9(8):e104966. doi: 10.1371/journal.pone.0104966 (PMC4133264; doi:10.1371/journal.pone.0104966)
Supplement: Table S1 — This table contains the 366 single nucleotide polymorphisms (SNPs) analyzed in our study. (DOCX) [file pone.0104966.s001.docx]

| Gene | Chr | Position | | Tag SNP ID |
| --- | --- | --- | --- | --- |
| Dkk-1 | 10 | 53743000..53748000 | | rs1569198, rs11001560 |
| GSK3 | 3 | 121016360..121307081 | | rs9813864, rs4688047, rs16830594, rs7431209, rs17811061, rs3732359 |
| NOG |  |  | | no HapMap Tag SNPs |
| Sclerostin | 17 | 39186000..39193000 | | rs851056, rs865429 |
| B-Catenin | 1 | 9827822..9896001 | | rs17034420, rs1220405, rs15177, rs2379107, rs1220412 |
| Alkaline Phosphatase | 1 | 21704993..21780942 | | rs1697404, rs12127343, rs4654755, rs12565845, rs4021228, rs10917008, rs6658127, rs1767429, rs2275370, rs1767430, rs6664097, rs3121206, rs6703976, rs3767152, rs1256341, rs2242420, rs12145027, rs975000 |
| LRP5 | 11 | 67829853..67980150 | | rs638076, rs923346, rs312023, rs3781579, rs627174, rs2508836, rs624947, rs314750, rs314773, rs648438, rs606989, rs312009, rs638051 |
| LRP6 | 12 | 12157656..12318315 | | rs7956548, rs17819999, rs11609634, rs7316466, rs17302049, rs7136900, rs2284396 |
| AXIN | 7 | 34589419..34589650 | | no reported tag SNPs |
| sFRP1 | 2 | 183403543..183443182 | | rs21714, rs2242070, rs288324, rs3768842, rs288316, rs4666865rs10206992 |
| Osteocalcin |  |  | | no tagSNPs with MAF > 0.1 |
| ANKH | 5 | 14762019..14924876 | | rs10054602, rs31916, rs31910, rs697569, rs153932, rs25987, rs2453327 rs706292, rs3087948, rs30613, rs4702049, rs27356, rs258229, rs2921600, rs16903723, rs744165, rs16903719, rs2291943, rs697571, rs3006069, rs10513191, rs31934, rs696294 |
| BMP15 | X | | 50670233..50676636 | rs12687730, rs3897937 |
| BMP2 | 20 | | 6696137..6709518 | rs235764, rs3178250 rs1005464, rs6054512 |
| BMP1 | 8 | | 22076518..22128127 | rs7819541, rs7592, rs7812993, rs13257482, rs4715, rs12675993, rs7827382, rs12114940, rs4075478, rs3857979, rs7814885, rs4342591, rs4242430, rs7838961, rs6983732 |
| BMP3 | 4 | | 82170013..82194878 | rs985328, rs1495634, rs10031561, rs10002189 |
| BMP4 | 14 | | 53486207..53491020 | rs17563, rs2071047 |
| BMP5 | 6 | | 55722189..55854340 | rs9296802, rs3807016, rs11537721, rs9475428, rs2397190, rs10456718, rs228144, rs3798843, rs10948955, rs17734678, rs9475432, rs16887173, rs1447129, rs9475394, rs10485072, rs9370471, rs228146, rs228141, rs228136, rs228143, rs3823038, rs9367669, rs228139, rs3798819, rs3798836, rs3798818 |
| BMP6 | 6 | | 7671304..7828115 | rs6938135, rs267195, rs270398, rs1235192, rs270388, rs11243204, rs267190, rs267806, rs4143048, rs11760020, rs408505, rs270378, rs911749, rs1150890, rs1225929, rs1225933, rs267802, rs2068361, rs1885448, rs267180, rs881891, rs11964227, rs592849, rs6910759, rs6910759, rs270406, rs11243205, rs1044104, rs2876117, rs932659, rs927406, rs7753111, rs267205, rs198354, rs2241669, |
| BMP7 | 20 | | 55174175..55279494 | rs6123669, rs162314, rs4811823, rs1015985, rs6127984, rs6127980, rs6064508, rs230202, rs162315, rs6025447, rs6123679, rs230188, rs6123674, rs3787380, rs6014967, rs230214, rs6127973 |
| BMP8 | 7 | | 34589419..34589650 | no Tag SNPs with MAF >0.1 |
| BMP10 | 2 | | 68945815..68952454 | rs2312078, rs2312076, rs3792229 |
| BMP11 | 2 | | 202945317..203145316 | rs2350809, rs6751210, rs4675278 |
| RUNX2 | 6 | | 45392894..45637933 | rs12208924, rs4510673, rs17209874, rs7750470, rs2064629, rs2396441, rs2790102, rs2677101, rs485817, rs6911631, rs16873396, rs2772395, rs2677108, rs6930053, rs1200425, rs12665622, rs9472487, rs17288341, rs6908650, rs9463090, rs4714860, rs12665523 |
| RANKL | 6 | | 149677598..149777597 | rs515133, rs237028, rs6570965, rs480670, rs11155639, rs576022, rs12665694, rs681919 |
| OPG (TNFRSF11B) | 8 | | 120003552..120034917 | rs3134061, rs11573829, rs2875845, rs3134058, rs3102724, rs11573897, rs11573856, rs3102728, rs1032128 |
| GDF5 | 20 | | 33484319..33489684 | rs224330, rs143384 |
| GDF15 | 19 | | 18357835..18361136 | rs1054564 |
| GDF9 | 5 | | 132224597..132228556 | rs10491279, rs17516673, rs254286 |
| GDF8 |  | |  | rs3791781 |
| GDF6 | 8 | | 97222815..97243118 | rs2514525, rs2440198 |
| GDF7 | 2 | | 20729664..20734971 | rs7589372 |
| GDF9B | 5 | | 132224597..132228556 | rs254286, rs17516673, rs10491279 |
| GDF10 | 10 | | 48045127..48059840 | rs9860, rs2853838, rs1902725, rs743509, rs1902724, rs2236724 |
| GDF11 |  | |  | rs11829536 |
| GDF1 | 19 | | 18838981..18869332 | rs4808868, rs2075762, rs7250622, rs11673194, rs726407, rs4808867, rs4808165, rs7259353 |
| GDF2 |  | |  | rs3781226, rs3740297 |
| GDF3 | 12 | | 7733350..7739925 | rs1123136, rs6488592, rs12819884, rs6488591, rs3815479 |
| WNT2 | 7 | | 116704518..116750579 | rs2285544, rs10487362, rs3729629, rs916725, rs733153, rs10227271, rs2896218, rs39312 |
| WNT3 | 17 | | 42196859..42251081 | rs11650531, rs7218567, rs10432043, rs2074405, rs2074404, rs199498, rs11079737, rs3851781, rs4968241, rs199520, rs199501, rs3916033, rs12452064, rs11658976 |
| WNT4 | 1 | | 22318787..22342197 | rs7526484, rs2235529, rs10917157, rs7544210, rs3765350 |
| WNT5 | 12 | | 1608673..1626638 | rs11061887, rs2240511, rs11061881, rs2240510, rs3803164, rs2270038, rs2270034, rs2270037, rs11061882, rs2240507 |
| WNT6 | 2 | | 219432790..219447198 | rs744092 |
| WNT7 | 22 | | 44696323..44751395 | rs10453447, rs28439308, rs9286452, rs9330811, rs10448605, rs10453441, rs9723264, rs10448592, rs10448585, rs10448600, rs10448607 |
| WNT7A | 3 | | 13835083..13896619 | rs6442416, rs4685039, rs11128662, rs1433353, rs17038684, rs4685041, rs6803033, rs1433355, rs3762721, rs9828013, rs12639607, rs13433875, rs17038710, rs9863149, rs12492784, rs7641735, rs12492620, rs7624679, rs41344949, rs11922919, rs9849631, rs734176, rs12634816 |
| WNT8 | 10 | | 102212802..102233387 | rs11190573, rs12762598 |
| WNT10 | 12 | | 47645391..47651810 | rs3741627, rs833843 |
| WNT10B | 2 | | 219453499..219466895 | rs10932786, rs7349332 |
| WNT11 | 11 | | 75575019..75595222 | rs948006, rs10899175, rs689095, rs94111, rs749311 |
| WNT16 | 7 | | 120752657..120768393 | rs3801387, rs2707466, rs3801385 |
| WIF1 | 12 | | 63730674..63801383 | rs6581606, rs12318609, rs3782498, rs7960504, rs7299766, rs2289936, rs1446527, rs7397906 |
| PSMB9 | 6 | | 32929632..32935887 | rs1351383, rs17587, rs991760, rs20547, rs2071535 |

366 single nucleotide polymorphisms (SNPs) within 52 genes were selected from public databases including the SNP database of the National Centre for Biotechnology Information (NCBI; http://www.ncbi.nlm.nih.gov/SNP/) and the International HapMap Project (http://www.hapmap.org/).
