# Supplementary material for: Bone Morphogenetic Protein 6 Polymorphisms Are Associated with Radiographic Progression in Ankylosing Spondylitis
Source: PLoS One. 2014 Aug 14;9(8):e104966. doi: 10.1371/journal.pone.0104966 (PMC4133264; doi:10.1371/journal.pone.0104966)
Supplement: Table S2 — SNPs Association between polymorphism associated with bone formation in AS patients (p<0.05, adjusted by age at onset of symptom, sex, disease duration, and smoking). (DOCX) [file pone.0104966.s002.docx]

| CHR | SNP | BP | A1 | OR | L95 | U95 | P | FDR thresholds |
| --- | --- | --- | --- | --- | --- | --- | --- | --- |
| 6 | rs270378 | 7762715 | C | 1.967 | 1.332 | 2.906 | 0.0006744 | 0.0015625 |
| 6 | rs1235192 | 7867046 | T | 0.5191 | 0.3494 | 0.7713 | 0.001171 | 0.001612903 |
| 6 | rs270388 | 7772340 | C | 1.855 | 1.252 | 2.748 | 0.002063 | 0.001666667 |
| 6 | rs270386 | 7773969 | C | 1.842 | 1.24 | 2.735 | 0.002477 | 0.001724138 |
| 8 | rs1032128 | 119951773 | G | 0.5692 | 0.3915 | 0.8275 | 0.003162 | 0.001785714 |
| 6 | rs1044104 | 7881311 | T | 0.5404 | 0.3588 | 0.8139 | 0.003228 | 0.001851852 |
| 6 | rs408505 | 7866427 | T | 0.5143 | 0.3298 | 0.8018 | 0.003338 | 0.001923077 |
| 8 | rs11573829 | 119959623 | A | 0.5333 | 0.3497 | 0.8131 | 0.003487 | 0.002 |
| 6 | rs270398 | 7765840 | G | 1.744 | 1.193 | 2.55 | 0.00407 | 0.002083333 |
| 6 | rs1226102 | 7763114 | G | 1.695 | 1.165 | 2.465 | 0.005779 | 0.002173913 |
| 6 | rs1225933 | 7876215 | T | 1.751 | 1.172 | 2.617 | 0.006288 | 0.002272727 |
| 6 | rs1150890 | 7797113 | T | 0.5424 | 0.3412 | 0.8623 | 0.009708 | 0.002380952 |
| 6 | rs1225929 | 7874233 | T | 0.5407 | 0.3387 | 0.8629 | 0.009938 | 0.0025 |
| 6 | rs1885448 | 7771778 | C | 1.607 | 1.11 | 2.326 | 0.01195 | 0.002631579 |
| 8 | rs3102724 | 119946807 | C | 0.6536 | 0.4605 | 0.9276 | 0.0173 | 0.002777778 |
| 1 | rs3121206 | 21907604 | C | 1.784 | 1.107 | 2.876 | 0.01752 | 0.002941176 |
| 6 | rs267180 | 7830141 | T | 1.593 | 1.081 | 2.348 | 0.01857 | 0.003125 |
| 6 | rs267802 | 7815519 | T | 1.665 | 1.089 | 2.545 | 0.01866 | 0.003333333 |
| 5 | rs2453327 | 14765106 | T | 1.587 | 1.079 | 2.335 | 0.01907 | 0.003571429 |
| 6 | rs267195 | 7847928 | T | 1.687 | 1.088 | 2.614 | 0.01934 | 0.003846154 |
| 6 | rs267205 | 7860317 | T | 0.6305 | 0.4224 | 0.9411 | 0.02401 | 0.004166667 |
| 6 | rs267190 | 7842121 | A | 1.612 | 1.058 | 2.454 | 0.02612 | 0.004545455 |
| 8 | rs3134058 | 119954108 | C | 0.6712 | 0.471 | 0.9566 | 0.02743 | 0.005 |
| 22 | rs28439308 | 46324625 | C | 1.531 | 1.038 | 2.26 | 0.03192 | 0.005555556 |
| 22 | rs10453447 | 46334133 | A | 1.541 | 1.037 | 2.291 | 0.03243 | 0.00625 |
| 1 | rs2242420 | 21904529 | T | 1.669 | 1.024 | 2.723 | 0.04001 | 0.007142857 |
| 6 | rs12665694 | 149672323 | C | 0.5631 | 0.3243 | 0.9777 | 0.04134 | 0.008333333 |
| 20 | rs143384 | 34025756 | C | 1.499 | 1.013 | 2.218 | 0.0427 | 0.01 |
| 2 | rs2312078 | 69095152 | G | 1.551 | 1.005 | 2.393 | 0.04759 | 0.0125 |
| 6 | rs267806 | 7819895 | G | 0.6474 | 0.4198 | 0.9984 | 0.04915 | 0.016666667 |
| 6 | rs198354 | 7817433 | G | 1.536 | 1.001 | 2.356 | 0.04926 | 0.025 |
| 6 | rs2068361 | 7736743 | A | 1.462 | 1.001 | 2.137 | 0.04967 | 0.05 |
